# Factor H Family Proteins in Complement Evasion of Microorganisms

**DOI:** 10.3389/fimmu.2017.00571

**Published:** 2017-05-18

**Authors:** Mihály Józsi

**Affiliations:** ^1^MTA-ELTE “Lendület” Complement Research Group, Department of Immunology, Eötvös Loránd University, Budapest, Hungary

**Keywords:** complement deregulation, complement evasion, microbial virulence, factor H, factor H-related, opsonization

## Abstract

Human-pathogenic microbes possess various means to avoid destruction by our immune system. These include interactions with the host complement system that may facilitate pathogen entry into cells and tissues, expression of molecules that defuse the effector complement components and complexes, and acquisition of host complement inhibitors to downregulate complement activity on the surface of the pathogen. A growing number of pathogenic microorganisms have acquired the ability to bind the complement inhibitor factor H (FH) from body fluids and thus hijack its host protecting function. In addition to FH, binding of FH-related (FHR) proteins was also demonstrated for several microbes. Initial studies assumed that these proteins are complement inhibitors similar to FH. However, recent evidence suggests that FHR proteins may rather enhance complement activation both directly and also by competing with the inhibitor FH for binding to certain ligands and surfaces. This mini review focuses on the role of the main alternative pathway regulator FH in host–pathogen interactions, as well as on the emerging role of the FHR proteins as enhancers of complement activation.

## Introduction

**Figure 1:**
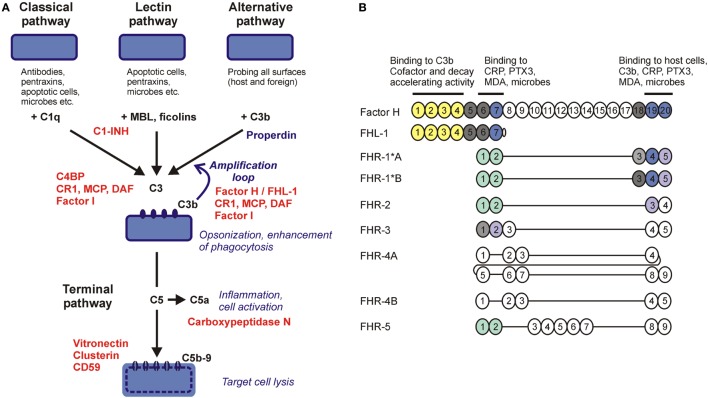
**Complement pathways and the human factor H (FH) protein family**. **(A)** Schematic overview of the major complement activation and regulation pathways. Molecules acting as complement inhibitors are shown in red. **(B)** The five human FH-related (FHR) proteins retained domains homologous to complement control protein domains 6–9 and 18–20 of FH (showed by vertical alignment). Colors indicate domains identical between FH and FHRs; light shades indicate high sequence similarity (>80% identity) but not complete identity. The domains marked green are closely related to each other but only distantly to FH and mediate dimerization of FHR-1, FHR-2, and FHR-5. Functional sites in FH are shown by horizontal lines. FH-like protein 1 (FHL-1) is a splice variant of FH.

Innate and adaptive immune mechanisms work in a collaborative manner to effectively eliminate invading microorganisms and develop immune memory. In turn, pathogenic microbes have acquired various means during their co-evolution with their host organisms to evade host immune responses. The complement system, a major humoral arm of innate immunity, includes ~40 plasma and cell membrane-anchored proteins that act in a cascade-like manner to opsonize microbes and facilitate their phagocytosis, activate cellular responses, initiate inflammation, or directly lyse certain microbes by punching holes into them (1). Complement can be activated by three major pathways, the classical, the lectin, and the alternative pathway. The recognition molecules of the pathways initiate activation by interacting with enzymatically active components that propagate the cascade and generate active complement fragments and complexes that mediate the biological effects of the system (Figure 1A) (2).

Because complement is a powerful system to facilitate destruction of microbes or other target cells, host cells and tissues are protected by various combinations of fluid phase and membrane complement regulatory proteins that fine tune and/or block the activation steps of the complement cascade, restrict activation in both time and space, and prevent the potential deleterious effects of full-blown, excessive activation (Figure 1A) (3). Most complement regulatory proteins are negative regulators, i.e., inhibitors of the various activation steps, including the soluble regulators C1-inhibitor, C4b-binding protein, factor H (FH), vitronectin and clusterin, and the membrane-anchored regulators complement receptor type 1, membrane cofactor protein, decay accelerating factor, and CD59. Properdin is a positive regulator of complement activation. Recently, the FH-related (FHR) proteins have emerged as additional positive regulators that promote activation of the system, particularly the alternative pathway (4, 5).

## The Human FH Protein Family

Factor H is a conserved plasma glycoprotein that inhibits the alternative pathway and the amplification loop (6, 7). By binding to C3b, a major cleavage product of the central complement component C3, FH prevents assembly of the C3bBb alternative pathway C3 convertase enzyme, facilitates the decay of the convertase if already formed by displacing bound Bb from C3b (decay accelerating activity), and acts as a cofactor for the plasma serine protease factor I that then cleaves C3b into the inactive form iC3b (cofactor activity). Interaction of FH with C3b also allows for regulating the C5 convertases.

Factor H is composed of 20 individually folding complement control protein (CCP) domains. The complement regulatory activities of FH are mediated by the N-terminal CCP1–4 domains, which harbor a C3b-binding site (8). CCP7 contains binding sites for certain ligands including glycosaminoglycans on host cellular surfaces, pentraxins, and malondialdehyde (MDA) epitopes generated by lipid peroxidation. The C-terminal CCP19–20 domains harbor binding sites for C3b/C3d, pentraxins, and sialic acid/glycosaminoglycans, and thus anchor FH on host surfaces under complement attack (i.e., with deposited C3b) (9–11). This allows FH for restriction of complement activation on host cells and also on non-cellular surfaces lacking membrane complement regulators, such as basement membranes. Thus, FH has an important function in self–non-self discrimination by recognizing specific host surfaces (12, 13).

The FH-like protein 1 (FHL-1) is derived from an alternative transcript of the *CFH* gene, and includes the seven N-terminal CCPs of FH plus four amino acids at its C-terminal end. FHL-1 shares with FH complement inhibiting and ligand-binding capacities associated with these domains but may display functional differences, as well, that need to be more precisely defined in the future (14).

In humans, five *CFHR* genes are found adjacent to the *CFH* gene and code for five distinct FHR proteins. These proteins have structural homology to FH; however, they lack domains homologous to CCPs 1–4 of FH that are responsible for the complement inhibiting activity (Figure 1B). Initial studies on FHRs investigated their complement inhibiting capacity, and some form of—generally weak—activity was indeed described for all of them. FHR-1 was reported to inhibit C5 and the terminal pathway (15), FHR-2 was to inhibit the alternative pathway C3 convertase and activation of the terminal pathway (16), FHR-3 and FHR-4 were to enhance the cofactor activity of FH (17), FHR-3 was also to possess cofactor activity on its own (18), and FHR-5 was to display weak cofactor activity and inhibit the C3 convertase in fluid phase (19). However, some of these reported activities were not confirmed by other studies, e.g., the terminal pathway inhibition by FHR-1 (20–22). In general, FHR proteins appear to lack significant complement inhibitory activity (4), but further studies are needed to clarify if any of the FHRs possess some form of such activity. Because FHRs were shown to interact with C3b, they may modulate C3b degradation by competing out FH, but may also interfere with the assembly and/or activity of the C3b containing convertase enzymes (i.e., the alternative pathway C3 convertase and the C5 convertases), as suggested for FHR-5 and FHR-2.

The conserved domains of the FHR proteins are homologous to CCPs 6–9 and 18–20 of FH (Figure 1B). Because CCPs 6–7 and 19–20 of FH mediate interactions of the complement regulator with C3b, the pentraxins C-reactive protein (CRP) and pentraxin 3 (PTX3), MDA epitopes, host cells, and basement membranes, due to the potentially overlapping ligand-binding capacity associated with the homologous domains, FHRs could interfere with FH functions through competition (23). Recent data suggest that, contrary to previous assumptions, a major role of the FHR proteins is to recognize and bind certain ligands, surfaces and cells, and thus act as competitive inhibitors of FH.

CCPs 1–2 of FHR-1, FHR-2, and FHR-5 were found to mediate dimerization of these proteins, thus increasing their avidity for surface-bound C3b and resulting in increased competition with FH, termed complement deregulation. Disease-associated mutants of these proteins with duplicated dimerization domains result in enhanced alternative pathway activation by diminishing FH binding to surface-bound C3b (21, 24, 25). FHR-5 can also compete with FH for binding to CRP, PTX3, and extracellular matrix, resulting in enhancement of complement activation (26). Altogether, these recent data support a major role for the FHRs in modulating alternative pathway activation as antagonists of FH.

In addition, FHR-4 was shown to activate the alternative pathway by binding C3b and allowing the assembly of an active C3bBb convertase, and also to promote classical pathway activation *via* its interaction with CRP (27–29). Similarly, FHR-5 was demonstrated to enhance alternative pathway activation by C3b binding (26).

**Figure 2:**
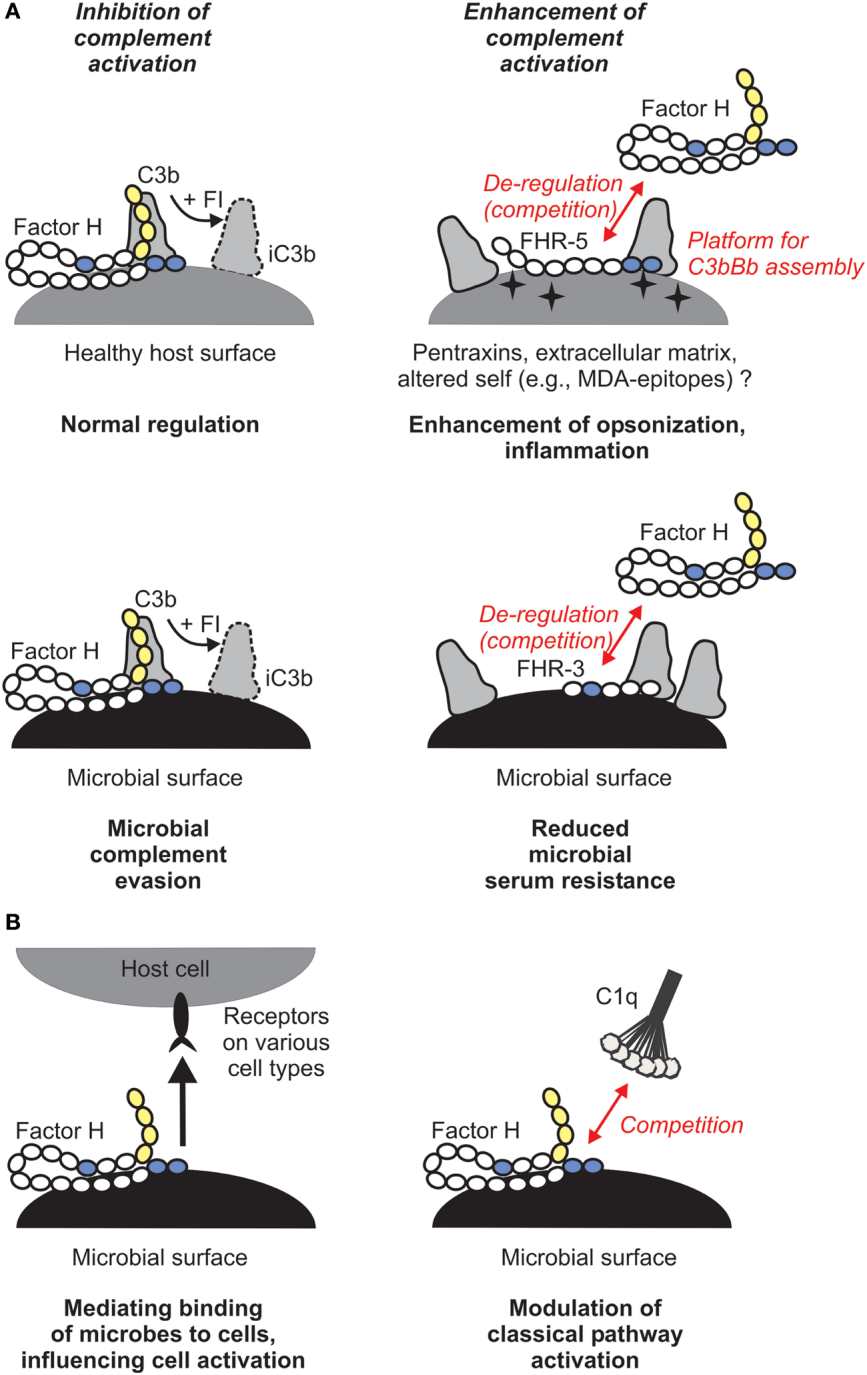
**Role of the human factor H (FH) protein family in microbial immune evasion**. **(A)** In addition to its role as plasma complement inhibitor, FH recognizes and binds to host surfaces and protects them from complement attack. Several microbes exploit this mechanism and recruit FH to their surface in order to escape from the complement system. FH-related (FHR) proteins may bind to certain host ligands or altered host surfaces that are exposed during inflammation or tissue damage (such as pentraxins, extracellular matrix proteins, or oxidative modifications of lipids) and displace FH, resulting in increased opsonization. FHRs may act as decoys and compete with FH for binding to microbial proteins. For example, FHR-3 was described to inhibit binding of FH to fHbp of *Neisseria meningitidis*. **(B)** FH was described to have additional functions. By simultaneously binding to certain microbes and receptors (such as CR3) on host cells, it may facilitate uptake of the microbe by immune cells and modulate cell activation, or facilitate entry of microbes into epithelial cells (left panel). FH was also shown to inhibit binding of C1q to apoptotic cells and *E. coli* and thus may modulate classical pathway activation and opsonization (right panel).

Why would this enhanced complement activation be useful for us as hosts and what does that mean in the context of infectious disease? Host FH is sequestered by pathogenic microbes, facilitating serum/complement resistance (Figure 2A). This can be an important step in evading first-line immune defense and aids dissemination of microbes and colonization of host niches. FHRs, in turn, were suggested to be decoys that due to their overlapping ligand spectrum with FH may displace this complement inhibitor from the surface of microbes, and may also fine tune complement activation under physiological conditions, e.g., on altered self (4). Thus, FHRs may increase opsonization of microbes, dying cells, and cellular debris, and help the resolution of inflammation (Figure 2A). Notably, most FH-binding microbial proteins also bind within those FH domains that are conserved among the FHR proteins (4, 30).

## Role of FH in Host–Microbe Interactions

Various classes of microbial pathogens were shown to bind human FH; these were reviewed in detail elsewhere [see, e.g., Ref. (30, 31)]. Instead of providing an ever-growing list of such microorganisms, this mini review aims to highlight general patterns (to which exceptions may exist) regarding the relevance of FH binding to microbes, and critically evaluate available literature, by discussing selected representative examples.

Overall, binding FH (or FHL-1) from body fluids is thought to be of advantage for pathogenic microbes in their survival in the host (Figure 2A). Prominent examples include the OspE protein of *Borrelia burgdorferi* (32), Sbi of *Staphylococcus aureus* (33), PspC of *Streptococcus pneumoniae* (34), and fHbp of *Neisseria meningitidis* (35). Sialylated *Neisseria gonorrhoeae* binds FH and provides an example of pathogen mimicry of host glycans (36, 31). Apparently, numerous and otherwise unrelated microbial proteins target the same conserved domains of FH, which thus involve pathogen- and host–ligand-binding sites. Such a common microbe binding site was determined and characterized in CCP20 of FH recently (37). Microbes thus can misdirect the self-recognition domains and mimic host ligands/surfaces (38).

Selective binding of FH is one of the reasons of host restriction of certain infections: human FH is preferentially bound by, e.g., group A streptococci (39), *N. meningitidis* (38, 40), *N. gonorrhoeae* (41), and non-typeable *Haemophilus influenzae* (42). By contrast, bacteria that infect various hosts, such as *B. burgdorferi*, bind FH from several species (43).

The importance of FH binding for bacterial survival is well documented for *N. meningitidis*, and fHbp is one of the components of *N. meningitidis* serogroup B vaccine (35, 38, 44, 45). In other cases, the role of FH as being beneficial for the microbe is controversial. The hypervariable region of several M proteins of *Streptococcus pyogenes* binds FH, which was attributed to downregulate opsonization and promote phagocytic resistance of the pathogen (46). Later studies, on the other hand, found no clear benefit of FH binding in resisting killing in a whole blood model or in an *in vivo* infection model (47). The used strains and models may influence this; recently, in a human FH transgenic mouse increased virulence of the *S. pyogenes* strain AP1 (which expresses protein H) was observed (39). Similarly, while several borrelial proteins with FH-binding capacity have been described (48), in some cases they may be dispensable for virulence (49).

Furthermore, some microorganisms were shown to degrade FH (50–52). This appears counterproductive because cleaved FH then loses its ability to inhibit complement activation (51). However, microbes may gain advantage from a more inflammatory micro-environment (53, 54) or, because their proteases could also cleave complement factors necessary for the propagation of the cascade (55), the functional inactivation of FH may not cause significant disadvantage in complement resistance. In addition, the kinetics of inactivation may allow sufficient regulation by FH. In any case, this issue needs further clarification.

Besides its role in the regulation of the alternative pathway, FH was also shown to compete with C1q for binding to lipid A component of LPS, and also to the surface of the *E. coli* strain TG1, pointing to the possibility that in certain cases FH may modulate the activity of the classical pathway (Figure 2B) (56, 57). This potentially important aspect needs to be further studied.

In addition, by binding to receptors on cells, FH can mediate microbe–host cell interactions (Figure 2B). In this non-canonical role, FH was described to act as a bridging molecule between complement receptor 3 (CR3; CD11b/CD18) and pathogens, and helping either pathogen entry into host cells or the antimicrobial response of the host cells (51, 58–62). Such scenarios were described for FH bound to *S. pneumoniae, N. gonorrhoeae*, and *Candida albicans* (58–61). FH bound on *C. albicans* was shown to facilitate the adhesion, phagocytosis and antifungal responses by neutrophilic granulocytes, such as increased lactoferrin and reactive oxygen species production (61). FH can also enhance the response of macrophages when exposed to C. *albicans* (51).

Thus, while for most studied microbes binding of FH (and in some cases also that of FHL-1) and the ability of FH/FHL-1 to act as a cofactor for C3b cleavage when bound on the surface or on certain microbial ligands were shown *in vitro*, direct evidence that demonstrates a relevant role of certain FH/FHR-binding proteins in serum resistance is less substantial. It is important to define the relative contribution of such potential virulence factors to microbial survival in serum and in animal models of infectious diseases. Novel technologies and model organisms may help clarifying to which extent specific FH-binding proteins contribute to the survival of pathogens. Studying non-pathogenic strains for FH binding and activity in parallel would likely be also informative.

## FHRs Binding to Microbes

Interaction of FHR proteins with microbes (63) has not yet been extensively studied; particularly, functional studies are scarce. This is related to our limited knowledge on these proteins, as discussed above. However, some important observations suggest that FHRs could emerge during evolution as decoys that counteract the sequestration of FH from host body fluids (4). Notably, in FHRs the conserved domains are homologous to those of FH that mediate binding of FH to various ligands/surfaces, both self and non-self, thus FHRs likely share the capacity to bind microbes. FHRs are also described in several non-human species, including mice, rats, and fish; these FHRs also lack the complement regulatory domains of FH and differ in number and domain composition from their human counterparts, there are no clear direct homologs (64–68).

In most cases, FHR-1 binding to microbes and microbial proteins that otherwise bind FH (and in some cases also FHL-1) was demonstrated, such as for several borrelial proteins (48, 69, 70), *Leptospira interrogans* (71), *S. aureus* (33), *Pseudomonas aeruginosa* (72), *N. gonorrhoeae* (60), *Plasmodium falciparum* (73), *C. albicans* (61), and *Aspergillus fumigatus* (74). So far, in most reports, no functional role for FHR-1 when associated/bound to microbes was demonstrated; in most cases, it was merely assumed that FHR-1 inhibits complement terminal pathway based on the report of Heinen et al. (15). In the case of the streptococcal Scl1 protein, FHR-1 was shown to inhibit terminal pathway activation (75). Even so, FHR-1 was shown not to influence bacterial opsonization and survival in the case of *B. burgdorferi* (70).

FHR-1, FHR-2, and FHR-5 bind to *B. burgdorferi*. Functional analysis, however, could not demonstrate a contribution of the FHR proteins to serum resistance of this microbe (70). On the other hand, FHR-1 bound on *C. albicans* was shown to facilitate interaction with human neutrophils and promote neutrophil antimicrobial responses (61).

*Fusobacterium necrophorum* binds FH, FHL-1, FHR-1, and FHR-4. Various strains were compared, and a weakly FH-binding strain showed increased C3b and terminal C5b-9 complex deposition on its surface, and decreased survival in human serum, compared with strains that bind FH stronger. The role of FHR-1 and FHR-4 was not addressed (76). FHR-4 also binds to *C. albicans*, but the functional relevance of this interaction is unclear (61). In both cases, the FHR-4A isoform (77) bound from serum, which shows increased C3b binding compared with FHR-4B, and activates the alternative pathway (29). Further studies need to assess its potential role in enhancing opsonization.

Direct evidence for an important role in infectious disease was described for FHR-3. A genome-wide disease-association study linked the *CFHR3* gene to *N. meningitidis* infection (78). A following functional study found that FHR-3 binds to this pathogen and competes with FH for binding to fHbp of *N. meningitidis*, thus acts as a competitive inhibitor of FH and enhances complement activation (Figure 2A). FHR-3 and FH bind with similar, nanomolar affinities to fHbp, but relative affinities differ between fHbp variants. Altogether, the genes of both the human host (by determining FH/FHR-3 levels) and the pathogen (by determining fHbp variants, e.g., that preferentially bind FH) influence disease susceptibility (79).

Additional indirect evidence supports such a role of the FHRs. For example, increased FHR concentrations were described in the middle-ear effusion fluid of patients with otitis media with effusion (80). In the zebrafish, FHR expression was found to be upregulated by LPS, indicating a role for them as acute phase proteins (68). These and other data (81) indicate that FHRs may be upregulated during infection or inflammation.

## Conclusion and Outlook

Although the role of FH in complement evasion is of medical importance for some microbes, further aspects of binding of this regulator need to be elucidated, such as the relevance of mediating cellular interactions and regulation of the classical pathway. The role of the FHR proteins is still poorly understood. While they emerge as positive complement regulators *via* competition with FH and by directly activating the alternative pathway through C3b binding, important questions include (1) the relative concentrations and their regulation, (2) affinity differences toward specific ligands, (3) functional redundancy among them, and (4) clarification of proposed and still unknown complement inhibitory capacity. Further studies will help to evaluate their role in host–pathogen interactions, identify novel vaccine candidates, and may also address the potential therapeutic use of FHR proteins in infectious diseases.

## Author Contributions

MJ prepared the text and the figures.

## Conflict of Interest Statement

The author declares that the research was conducted in the absence of any commercial or financial relationships that could be construed as a potential conflict of interest.
